# Surgical Versus Conservative Management of Delayed Presentation of Acute Biliary Disease: A Systematic Literature Review

**DOI:** 10.7759/cureus.74237

**Published:** 2024-11-22

**Authors:** Ahmad Khalifa, Sajad J Allami, Owais Tahhan, Shaikha S Alhaj, Mohamad A Al Tahan, Ibrahim Elnogoomi

**Affiliations:** 1 Surgery, University of Aleppo Medical College, Aleppo, SYR; 2 Surgery, University of Sharjah, Sharjah, ARE; 3 Urology, Sandwell and West Birmingham Hospitals NHS Trust, Birmingham, GBR; 4 Graduate Medical Education, Dubai Health Care, Sharjah, ARE; 5 Pharmacy, Aston University, Birmingham, GBR; 6 Surgery, Kuwait Hospital, Sharjah, ARE

**Keywords:** acute presentation, antibiotic administration in management of severe sepsis, biliary, cholecystectomy, conservative vs surgical management, delayed presentation, laparoscopic, patient outcomes, sepsis, systemic review

## Abstract

Biliary sepsis, characterized by contamination and infection of the biliary tract, poses a serious medical issue with detrimental effects on the patients. While cholecystectomy is the usual treatment for symptomatic gallstones, the most desirable management approach for biliary sepsis remains debated, prompting a scientific evaluation of the long-term effects of cholecystectomy. To compare the long-term outcomes of biliary sepsis in patients undergoing cholecystectomy versus conservative management (CM), this study will systematically review the existing literature to clarify differences in recurrence rates, complication rates, and overall survival. PubMed and the Cochrane Library were searched thoroughly for the literature review. Studies were included if they reported the effects of surgical and conservative interventions on predefined patient outcomes. A critical appraisal of the studies included was performed using CASP criteria. Fourteen studies were included, comprising prospective cohort studies and randomized controlled trials, with sample sizes varying from 52 to 234 patients. Endoscopic sphincterotomy (ES), early versus delayed laparoscopic cholecystectomy (D-LC), combined endoscopic-laparoscopic techniques, and percutaneous cholecystostomy followed by early laparoscopic cholecystectomy (E-LC) were the analyzed interventions. The primary conclusions showed that, in comparison to D-LC, E-LC significantly reduced hospital stays (p < 0.05), since the times were 58 and 167 hours for E-LC and D-LC, respectively. Additionally, E-LC resulted in fewer recurrent biliary events (4.3 compared to 36.2% of D-LC) and lower overall costs. ES demonstrated efficacy in mitigating the requirement for emergency cholecystectomy in patients at high risk, as evidenced by its 94% success rate in endoscopic stone removal. Without increasing postoperative complications, combined endoscopic-laparoscopic techniques showed high success rates for stone removal (95.6% common bile duct clearance rate). This systematic review highlights the favorable long-term effects of cholecystectomy in managing biliary sepsis. It emphasizes the importance of individualized treatment processes and considers conservative control for patients with high surgical risk and significant comorbidities. It also highlights the need for advancement in CM and provides insights that can help clinical decision-making to optimize outcomes in affected patients.

## Introduction and background

Managing cholelithiasis or gallstone sickness strains healthcare structures globally, with costs achieving about $6.5 billion annually within the USA [1]. Gallstone prevalence varies from 0.1% to 50.5% [2]. Even though the disease can be asymptomatic, some factors could necessitate treatment [3]. Gallstone-related complications include common bile duct (CBD) stones (choledocholithiasis), acute cholecystitis, cholangitis, gallstone pancreatitis, and sometimes cholangiocarcinoma [4,5]. Surgical removal of the gallbladder, or cholecystectomy, is the preferred treatment for symptomatic gallstones [6]. Laparoscopic cholecystectomy (LC) is favored over open cholecystectomy for treating gallstones and cholecystitis [7]. However, around 12% of patients who undergo cholecystectomy continue to experience pain and recurrent gallstone-associated symptoms [8]. Conservative management (CM), which involves treating symptoms and pain without removing the gallbladder, avoids the risks associated with surgery and is considered an alternative to cholecystectomy [9]. However, for patients with uncomplicated gallstones, there is often a recurrence of symptoms and the development of gallstone-related complications, eventually necessitating cholecystectomy [10]. Several randomized controlled trials (RCTs) comparing early versus delayed cholecystectomy (DC) for gallstone disease have reported that delaying surgery leads to the recurrence of symptoms during the conservative treatment period, as well as higher post-surgical complication rates [11]. Amongst the serious conditions that result from complications is biliary tract sepsis, which is often associated with significant benign or malignant diseases of the biliary tract, pancreas, and hepatic hilus [12]. Determining the appropriate treatment approach - whether medical therapy, percutaneous/endoscopic intervention, or surgical treatment - can be challenging [13]. For patients with benign biliary tract stenosis, percutaneous drainage is typically the first-line treatment, while surgery is reserved for cases where bilioplasty is unsuccessful or for segmental extrahepatic sclerosing cholangitis [14].

Rationale

Cholecystectomy is the usual intervention for preventing recurrent biliary complications. Yet, a subset of patients continues to face unfavorable outcomes despite undergoing surgery. On the contrary, conservative control, which avoids surgical risks, may also result in recurrent signs and complications eventually necessitating surgical intervention. The management of biliary sepsis, a severe and potentially life-threatening condition, is a major challenge in medical practice highlighting the need to identify the most effective treatment strategies. Given the major healthcare burden and the potential for the development of severe complications, a systematic comparison is critical for assessing the long-term outcomes of biliary sepsis among different treatment cohorts, conservative versus cholecystectomy groups. While several previous reviews of the literature exist, no prior study has compared such a broad spectrum of surgical interventions with CM. This review aims to address this gap by providing a thorough evaluation of the available evidence.

## Review

Objectives

The aim of this study is to systematically evaluate the existing literature to analyze the long-term outcomes of biliary sepsis in two distinct patient cohorts: patients undergoing cholecystectomy and cases managed conservatively without surgical intervention. The study will compare the two management approaches (cholecystectomy versus CM) in terms of recurrence rates of biliary sepsis, complication rates, overall survival following episodes of cholecystitis and cholangitis sepsis, cost-effectiveness, and length of hospital stay. Additionally, the study will assess the safety and effectiveness of cholecystectomy compared to CM in reducing the risk of recurrent biliary complications and improving overall patient outcomes.

Materials and methods

Study Design

A systematic review was performed according to the Preferred Reporting Items for Systematic Review and Meta-Analysis (PRISMA) [15]. Pre-existing data was used in this study, and, therefore, ethical approval is not applicable.

Search Strategy

A comprehensive literature search using PubMed and the Cochrane Library electronic databases was conducted by three independent researchers from 1990 to 2024. Boolean operators and multiple filters were applied to refine the research strategy. All relevant articles investigating surgical and CM of patients with biliary tract infections were retrieved and duplicate studies were removed. A detailed search strategy used to retrieve studies from both databases is given in Table 1.

**Table 1:** Search strategy for the systematic review and meta-analysis (SRMA)

Database	Search String	Results
PubMed	("Sepsis"[MeSH] OR "Biliary Tract Infections"[MeSH] OR "biliary sepsis" OR "biliary tract sepsis" OR "biliary infections and sepsis") AND ("Cholecystectomy"[MeSH] OR "surgical removal of gallbladder" OR "gallbladder surgery") AND ("Watchful Waiting"[MeSH] OR "Conservative Treatment"[MeSH] OR "non-surgical management" OR "conservative treatment" OR "medical management" OR "non-operative treatment") AND ("Cholecystitis"[MeSH] OR "gallbladder inflammation" OR "acute cholecystitis") AND ("Cholangitis"[MeSH] OR "bile duct inflammation" OR "acute cholangitis")	226
Cochrane Library	("Treatment Outcome" OR "long-term outcomes" OR "long-term follow-up" OR "outcomes") AND ("Aged" OR "Geriatrics" OR "elderly" OR "older adults" OR "geriatric patients" OR "aged") AND ("Cholecystectomy" OR "non-operative management" OR "deferred cholecystectomy" OR "delayed surgery" OR "no early cholecystectomy" OR "conservative management") AND ("Cholangiopancreatography, Endoscopic Retrograde" OR "ERCP" OR "endoscopic retrograde cholangiopancreatography") AND ("Sphincterotomy, Endoscopic" OR "sphincterotomy" OR "endoscopic sphincterotomy") AND ("Choledocholithiasis" OR "common bile duct stones" OR "CBD stones" OR "bile duct stones")	89

Eligibility Criteria

**Figure 1:**
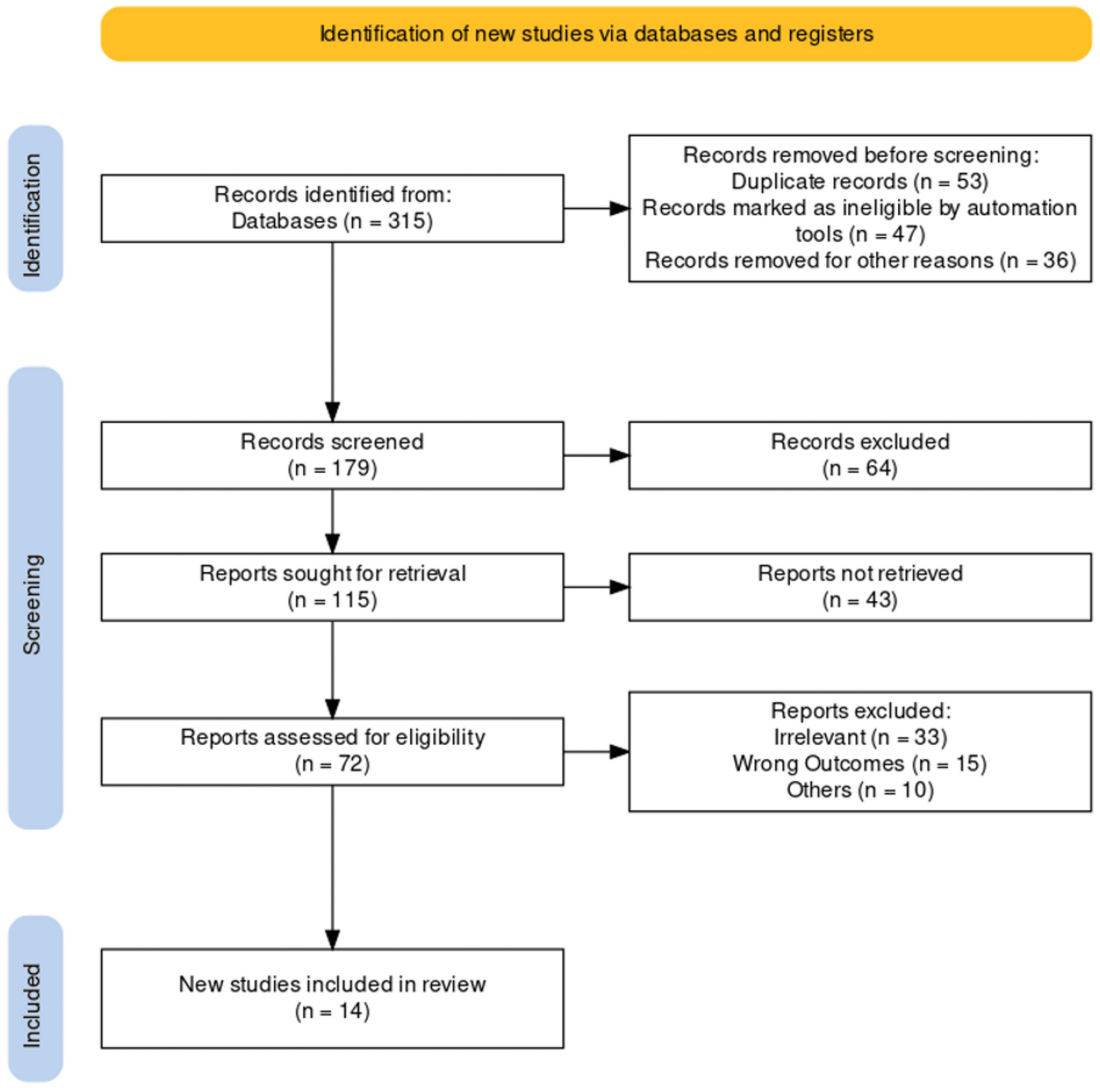
PRISMA flow chart of included studies PRISMA: Preferred Reporting Items for Systematic Review and Meta-Analysis

The study selection and inclusion process adhered to PRISMA guidelines, as detailed in a PRISMA flowchart (Figure 1). This chart illustrates our systematic approach to identifying, screening, and including studies, ensuring methodological transparency. Studies were assessed based on a predetermined inclusion and exclusion criteria framework, focusing on patient population, type of intervention, and relevance to biliary sepsis management outcomes.

The inclusion criteria for this review are studies reporting on patients diagnosed with biliary sepsis following both acute cholecystitis and cholangitis; studies evaluating long-term results (assessed at least six months post-treatment) in patients who underwent cholecystectomy for biliary sepsis versus those managed conservatively without surgical intervention; studies reporting on the recurrence of biliary complications, such as recurrent cholecystitis, cholangitis, and common bile duct stones, as well as treatment-related complications, including postoperative issues and complications from conservative management, and overall survival; studies published in English; randomized controlled trials (RCTs), cohort, case-control, and retrospective studies; studies conducted on adult populations aged 18 years and above; and studies with full-text availability.

The exclusion criteria for this review include studies that do not specifically mention biliary sepsis or do not clearly identify biliary sepsis as the primary condition; studies in which the main intervention is neither cholecystectomy nor conservative management for biliary sepsis; studies with insufficient reporting on long-term effects, inadequate statistics on recurrence and complication rates, overall survival, or those measuring outcomes unrelated to the prespecified objectives; studies published in languages other than English due to limited translation resources; case reports, letters, editorials, conference abstracts, and review articles lacking unique records; and studies conducted exclusively on pediatric populations (under 18 years of age).

Selection Process

In accordance with the inclusion criteria, a group of three researchers searched for studies based on their titles and abstracts. Additionally, publications were hand-searched in peer-reviewed journals with high-impact factors such as *Gastroenterology*, *Ann Surg*, *World J Gastroenterol*, *HPB*, *Am J Gastroenterol*, *World J Surg*, *Brit J Surg*, and *Dig Surg* to reduce the risk of publication bias. All selected studies were uploaded to Rayyan screening software for primary and secondary literature screening. Full texts of the articles that satisfied the inclusion criteria were thoroughly assessed. Studies that did not meet the eligibility criteria were excluded and any discrepancies were resolved by consensus. Study characteristics such as name of the author, publication year, treatment regimen in intervention and control, primary and secondary outcomes, and adverse events were extracted from the studies. All the included studies were thoroughly assessed to retrieve relevant data items.

Risk of Bias Assessment

We used a modified version of the Critical Appraisal Skills Programme (CASP) criteria developed by Guyatt et al. [16] to assess the methodological quality of the included studies. CASP criteria include 11 specific questions designed to assess the quality of each study. The criteria included three broad categories of questions as follows: (1) Were the findings of the study validated? (2) What were the results? (3) Are the findings of the study applicable locally? Researchers answer these questions with "YES" or "NO," and the number of "YES" responses out of the overall 11 questions represents the score for each study. If the first question was answered with a "Yes," then the remaining questions were answered the same. An overlap in the questions occurred, and the explanations for the answers and comments from researchers have been included.

Individual Study Analysis

This systematic review was conducted using individual studies rather than a combined meta-analysis. This approach was chosen to retain the unique outcomes and study designs of each selected work, which varied in terms of interventions, patient demographics, and clinical settings. Pooling data was avoided due to heterogeneity across studies, particularly in intervention timelines, outcome measures, and patient populations. Studies were selected based on their relevance to key outcomes of interest, such as recurrence rates, length of hospital stay, and complication rates, as well as methodological rigor and sample size. To provide a detailed assessment of clinical outcomes across different contexts, studies like Akyürek et al. [17] and Reinders et al. [18] were chosen to represent varying approaches to managing high-risk surgical patients with biliary sepsis. For example, Akyürek et al. provide insights into early LC outcomes following percutaneous cholecystostomy, while Reinders et al. focus on the timing of LC following ES. Each study’s specific design allows for a nuanced discussion of recurrence rates, sepsis risk, and LOS, which is central to understanding the clinical impact of early versus delayed intervention.

Results

Literature Search and Study Characteristics

A total of 315 studies were retrieved after a comprehensive search from PubMed and the Cochrane Library. Studies were also selected from journals and other independent resources if the reports were available. After removing duplicates and intensive screening according to the eligibility criteria, a total of 14 studies [17-30] were included in our systematic review. The synthesis of our findings of the included 14 studies is illustrated in Table 2.

**Table 2:** Synthesis table of included studies PC: percutaneous cholecystostomy; LC: laparoscopic cholecystectomy; DLC: delayed laparoscopic cholecystectomy; ERCP: endoscopic retrograde cholangiopancreatography; ES: endoscopic sphincterotomy; MGP: mild gallstone pancreatitis; E-LC: early laparoscopic cholecystectomy; D-LC: delayed laparoscopic cholecystectomy; CBD: common bile duct; BDE: bile duct exploration; CBDS: common bile duct stones; GBS: gallbladder stones; IOES: intraoperative endoscopic sphincterotomy; LCBDE: laparoscopic common bile duct exploration; LBDE: laparoscopic bile duct exploration; POS: postoperative stay; CCI: comprehensive complication index

Sr No.	Study	Location	Study design	Sample size	Population	Intervention	Comparison	Main findings
1	Akyürek et al. 2005 [17]	Turkey	Randomized clinical trial	61	High-risk patients with acute calculous cholecystitis	Percutaneous cholecystostomy (PC) followed by early laparoscopic cholecystectomy (LC)	Conservative treatment followed by delayed LC	In the PCLC group, all patients had symptom relief within 24 hours, and 29 (93.5%) of the 31 patients who had early LC attempted to do so after their clinical condition had stabilized enough. Thirty patients in the DLC group attempted delayed LC, and 26 (86.6%) of them were successful. The PCLC group experienced a shorter hospital stay and a lower cost compared to the DLC group.
2	Vracko et al. 2006 [18]	Sweden	Prospective cohort study	105	105 patients over 65 years of age (52 men, 53 women; mean age 78) suffering from acute cholecystitis were initially treated on a random basis with either conservative methods or endoscopic sphincterotomy	Endoscopic sphincterotomy	Conservative treatment	Following endoscopic sphincterotomy, three patients experienced iatrogenic complications; two of them were treated conservatively, and one required surgery. In 48 patients receiving endoscopic sphincterotomy and 36 patients receiving conservative treatment, the clinical course improved, preventing the need for emergency cholecystectomy and other interventions (p < 0.01).
3	Reinders et al. 2010 [19]	Netherlands	Randomized clinical trial	96	Patients with cholecystocholedocholithiasis who underwent successful ES	Early laparoscopic cholecystectomy (LC)	Delayed LC	The conversion rate (4.3% in the early group vs. 8.7% in the delayed group) and operating times, challenges, and hospital stays did not differ between the groups. One patient in the early group and patients in the delayed group (36.2%) experienced recurrent biliary events during the LC waiting period (p < 0.05).
4	Riquelme et al. 2019 [20]	Chile	Randomized controlled trial	52	All patients aged 18-70 admitted between January 2016 and October 2017 with first episode of mild gallstone pancreatitis (MGP)	Early laparoscopic cholecystectomy (LC)	Delayed LC	52 patients (26 E-LC, 26 D-LC) were randomized at the time of the interim analysis. A significantly shorter length of stay (median 58 versus 167 h; p < 0.05) was linked to E-LC as opposed to D-LC. Between the two methods, there were no differences in the need for an ERCP for choledocholithiasis (E-LC 26.9% versus D-LC 23.1%, p = 1.00).
5	Poh et al. 2018 [21]	Australia	Prospective cohort	104	Patients with choledocholithiasis	Emergency laparoscopic cholecystectomy with serum total bilirubin of below 50	Emergency laparoscopic cholecystectomy with serum total bilirubin of above 50 (jaundice group)	The jaundiced group's overall morbidity rate was 28 compared to 36% in the control group (p = 0.405). The control group's mean CCI was 8.00, while the jaundiced group was 5.28 (p = 0.229). The jaundiced group's mean length of stay was 4.65 days as opposed to 6.51 days (p < 0.05). There were neither conversions to open surgery nor peri-operative deaths.
6	Iodice et al. 2001 [22]	Italy	Clinical trial	52	52 patients (mean age 57.0 years; age range 20 to 89 years; 35 women and 17 men) with gallbladder and BD stones	Combined endoscopic-laparoscopic technique	NA	In 94% of cases, endoscopic stone removal was successful and did not result in any ERCP or surgical complications. The length of the operation was approximately 20 minutes longer, but the length of hospital stay was the same as that of a straightforward laparoscopic cholecystectomy.
7	El Nakeeb et al. 2015 [23]	Egypt	Prospective randomized study	110	Patients had CBDS with gallbladder stones (GBS)	Early LC within three days of endoscopic retrograde cholangiopancreatography (ERCP)	Delayed LC, one month after early versus late cholecystectomy (ERCP)	Regarding the conversion rate, degree of adhesion, cystic duct diameter, and intraoperative common bile duct injury or bleeding, there was no discernible difference between the two groups. Recurrent biliary symptoms were found in 7 (12.71%) of the delayed LC patients compared to 1 patient in the early LC group (p < 0.05).
8	Liu et al. 2020 [24]	China	Cohort study	207	Bile duct stones in patients with cholelithiasis	Laparoscopic cholecystectomy (LC) combined with common bile duct exploration	LC combined with endoscopic stone extraction	Patients with impacted stones at the end of the common bile duct (CBD) and those with stenosis of the sphincter of Oddi did not respond well to the LC + BDE with primary closure procedure. Stone location was the only variable that showed a significant difference between these two treatment groups. Other than stone size, CBD size, and location, there were no appreciable differences between the two groups in terms of these variables.
9	Morino et al. 2006 [25]	Italy	Prospective, randomized trial	91	Patients with gallbladder and bile duct stones	Preoperative endoscopic retrograde cholangiopancreatography (ERCP) with endoscopic sphincterotomy (ES) followed by LC	LC associated with intraoperative ERCP and ES	Group I had an 80% CBD clearance rate, while Group II had a 95.6% CBD clearance rate (p = 0.06). Group I had a morbidity rate of 8.8%, while group II had a rate of 6.5% (p = 1.00). In neither group were there any deaths. Group II's hospital stay was shorter than group I's, lasting 4.3 days as opposed to 8.0 days (p < 0.05).
10	ElGeidie et al. 2011 [26]	Egypt	Prospective randomized trial	226	Patients with common bile duct stones	Laparoscopic cholecystectomy (LC) combined with intraoperative endoscopic sphincterotomy (IOES)	LC with laparoscopic common bile duct exploration (LCBDE)	Between the two interventions, there was no statistically significant difference in the success rate of CBD clearance (92% for LC-LCBDE vs. 97.2% for LC-IOES, p = 1.00).
11	Poh et al. 2016 [27]	Australia	Randomized clinical trial	52	Patients with choledocholithiasis	Emergency laparoscopic cholecystectomy with intraoperative endoscopic retrograde cholangiopancreatography (ERCP)	Emergency laparoscopic cholecystectomy with laparoscopic bile duct exploration (LBDE)	The percentage of patients who had duct clearance was 87% for those who underwent intraoperative ERCP, and 69% for those in the LBDE group (p = 0.057). Retained stone rates were lower in the ERCP group (15%) than in the LBDE group (42%), respectively(p < 0.05).
12	Hong et al. 2006 [28]	China	Clinical trial	234	Patients with cholecystocholedocholithiasis	Laparoscopic cholecystectomy combined with intraoperative laparoscopic exploration of the common bile duct	Laparoscopic cholecystectomy and intraoperative endoscopic sphincterotomy	Regarding surgical time, success rate, number of stone extractions, postoperative complications, retained common bile duct stones, length of stay following surgery, and hospital charge, there were no differences between the two groups.
13	Bansal et al. 2014 [29]	USA	Randomized controlled trial	168	Patients with concomitant gallbladder stones and common bile duct stones	Single-stage laparoscopic common bile duct exploration and cholecystectomy	Endoscopic retrograde cholangiopancreatography (ERCP) for endoscopic extraction of CBD stones followed by LC	The two groups were matched based on clinical and demographic characteristics. Laparoscopic CBD exploration and ERCP for CBD clearance had comparable success rates (91.7% vs. 88.1%). Additionally comparable was the overall success rate, which was 78.8% in group 2 and 88.1% in group 1 (p = 0.20).
14	Noble et al. 2009 [30]	UK	Randomized clinical trial	91	Higher-risk patients with choledocholithiasis	Endoscopic sphincterotomy and subsequent laparoscopic cholecystectomy (LC)	Laparoscopic bile duct exploration (LBDE) during LC	In terms of intention-to-treat, 29 out of 47 patients in Group A and 44 out of 44 patients in Group B had duct clearance (p < 0.05). Eight of the 47 and eight of the 44 patients (p ¼ 0.884) experienced Clavien Grade II-V complications; the median number of procedures performed was 2 (2-3) and 1 (1-1) (p < 0.05); 2 of the 47 and 4 of the 44 patients needed conversion (p = 0.676); and the median postoperative stay (POS) was 3 (2-7) and 5 (2-7) days (p = 0.825), respectively.

Risk of Bias Assessment

Our findings, including those on LOS, postoperative complications, and recurrence rates, are presented based on individual study results rather than pooled estimates. For instance, we report length of stay outcomes from Riquelme et al. [20], who examined early LC, separately from Poh et al. [21], who explored jaundice-related risks, to underscore specific study contexts and patient subgroups. This method highlights the varied clinical settings where each study was conducted, facilitating a comprehensive assessment of each intervention’s impact. Where possible, similar outcomes across studies are presented together to demonstrate consistency or divergence. For example, Akyürek et al. [17], Riquelme et al. [20], and El Nakeeb et al. [23] collectively suggest that early LC generally reduces length of stay compared to delayed approaches, despite differences in study populations and intervention timing. This layered approach enables a more nuanced understanding of each study’s contribution to overall trends in biliary sepsis management.

As stated earlier, the CASP device was used to assess the quality of all the studies included in our systematic review. A CASP device was used to create the assessment table for all the studies included in our review. In this review, changes to the CASP appraisal screening questionnaire were made to streamline the appraisal method. The objective was to simplify the assessment method and make it less difficult to discover any methodological flaws within the studies. An overlap in the questions occurred, and the explanations for the answers and comments from researchers have been included. The risk of bias for the 14 included studies is shown in Table 3.

**Table 3:** CASP (modified LOW appraisal criteria) analysis CASP: Critical Appraisal Skills Programme; LOW: levels of evidence and weight

Questions	Akyürek et al. 2005 [17]	Vracko et al. 2006 [18]	Reinders et al. 2010 [19]	Riquelme et al. 2019 [20]	Poh et al. 2018 [21]	Iodice et al. 2001 [22]	Nakeeb et al. 2015 [23]	Liu et al. 2020 [24]	Morino et al. 2006 [25]	ElGeidie et al. 2011 [26]	Poh et al. 2016 [27]	Hong et al. 2006 [28]	Bansal et al. 2014 [29]	Noble et al. 2009 [30]
Did the study address a clearly focused question?	Y	Y	Y	Y	Y	Y	Y	Y	Y	Y	Y	Y	Y	Y
Did the authors look for the right type of papers?	Y	Y	Y	Y	Y	Y	Y	Y	Y	Y	Y	Y	N	N
Do you think all the important, relevant studies were included?	Y	Y	Y	?	N	Y	Y	Y	Y	N	N	Y	N	?
Did the review’s authors do enough to assess the quality of the included studies?	Y	?	Y	?	N	N	Y	Y	Y	Y	N	N	N	N
If the results of the review have been combined, was it reasonable to do so?	Y	Y	Y	N	N	Y	N	N	N	Y	Y	Y	Y	Y
Have the authors taken account of the potential confounding factors in the design and/or in their analysis?	?	N	Y	N	N	N	Y	Y	Y	N	N	?	?	N
Are the results clear to the reader?	Y	Y	?	Y	Y	Y	Y	Y	Y	Y	Y	Y	Y	Y
Are the results precise?	Y	?	N	N	Y	N	Y	Y	Y	N	N	N	N	N
Is the model validated?	?	Y	N	N	Y	N	Y	Y	Y	Y	Y	Y	N	N
Is the model applicable to a general population?	N	?	Y	N	Y	N	Y	Y	N	N	N	Y	Y	Y
Do the results fit with other available evidence?	Y	Y	Y	Y	Y	Y	Y	Y	Y	Y	Y	Y	Y	Y
Score out of 11	8	7	8	4	7	6	10	10	9	7	6	8	5	5

Summary of Evidence

Sepsis in patients at high surgical risk can be resolved with PC. Once these patients' acute infection and sepsis had cleared up, early LC could be safely carried out [17]. Most elderly patients with acute cholecystitis improved clinically after ES, indicating that biliary sepsis risk is lowered when obstruction at the common sphincter is relieved early [19]. A study showed that 36.2% of patients whose LC was postponed for 6-8 weeks experienced recurrent biliary events in a randomized trial assessing the timing of LC following ES. Early LC (within 72 hours) seems safe and may be able to avoid most biliary events during this time after sphincterotomy [18]. The E-LC strategy did not result in clinically significant postoperative complications and dramatically decreased the length of stay in patients with mild gallstone pancreatitis (MGP) [20]. When patients undergoing LC are diagnosed with choledocholithiasis, the presence of jaundice does not seem to be associated with a higher risk of complications [21]. The combined endoscopic laparoscopy approach is a safe and efficient way to treat CBD stones and gallbladder stones simultaneously [22]. There was no case converted to open cholecystectomy in either ERCP or LC, highlighting the efficacy of combined intervention over ERCP before LC reducing the length of hospital stay and associated cost in patients with gall bladder and bile duct stones. While waiting for LC, recurrent biliary symptoms were considerably more common in delayed LC patients. Significantly higher morbidity was seen in delayed LC [23]. Compared to the LC + endoscopic stone extraction (ESE) procedure, LC + common bile duct exploration (BDE) with primary closure was a safer and more effective way to treat cholecystitis and common bile duct stones (CCBDS) patients. It was also not linked to any increased risk of complications related to the T-tube or sphincterotomy of the duodenal papilla (EST) [24]. The laparoendoscopic rendezvous approach offers a higher rate of CBD stone clearance, shorter hospital stays, and lower costs when compared to preoperative ERCP with ES followed by LC [25]. For cholecystocholedocholithiasis, both LC combined with intraoperative ES (LC-IOES) and LC with laparoscopic common bile duct exploration (LC-LCBDE) have been demonstrated to be safe, effective, minimally invasive treatments; however, the former may be chosen in situations where endoscopic therapy resources and expertise are available [26]. Regarding reducing the incidence of retained stones in choledocholithiasis patients undergoing emergency LC, intraoperative ERCP is superior to laparoscopic bile duct exploration (LBDE) [27]. It has been demonstrated that LC-IOES and LC-LCBDE are both low-risk, low-invasive treatments for cholecystocholedocholithiasis [28]. Regarding surgical time, success rate, number of stone extractions, postoperative complications, retained CCBDS, length of stay following surgery, and hospital charge, there were no differences between the two groups [29]. Regarding postoperative stay, complications, or conversion in patients at higher risk, there was no difference between the approaches used for duct clearance; however, the laparoscopic approach was more successful and efficient and avoided needless procedures [30]. When interpreting the findings of research on surgical treatments for gallbladder and bile duct disorders, confounding variables are crucial. The severity of the initial condition and the timing of intervention, for example, are potential confounders in Akyürek et al. [17] and Reinders et al. [18], as patients with more severe symptoms or varying levels of clinical stability may react differently to early versus delayed LC. Similarly, Vracko et al. [19] emphasized that key confounding variables that could impact the clinical course and efficacy of conservative treatment versus ES included age, comorbidities, and the severity of cholecystitis. The existence of jaundice and the features of bile duct stones, respectively, were important confounders that could affect procedural success and morbidity rates in studies such as Poh et al. [21] and Liu et al. [24]. In trials by Morino et al. [25] and ElGeidie et al. [26], the initial clinical condition and preoperative management strategies were important confounders because they could cause variations in CBD clearance rates and postoperative outcomes. Furthermore, research by El Nakeeb et al., Poh et al., and Hong et al. shows that the timing of intervention and initial condition were recurrent confounders in studies comparing early versus late cholecystectomy or different endoscopic techniques [23,27,28]. It is crucial to take these confounding variables into account when interpreting study results. It makes sure that variations in results are not due to different clinical management approaches or underlying patient characteristics, but rather to the interventions themselves. Further research is warranted to account for these variables via meticulous study planning and statistical modifications to offer more precise perspectives on the efficiency and safety of various surgical procedures for gallbladder and bile duct disorders.

Long-Term Sepsis Recurrence and Incidence

According to Akyürek et al., early LC following PC led to a lower risk of sepsis recurrence, shorter hospital stays, and lower costs than delayed LC [17].

When comparing patients treated with ES to conservative treatment, Vracko et al. found no recurrence of sepsis, indicating significantly improved clinical outcomes (p < 0.01) [19]. In contrast to delayed LC, early LC (within 72 hours) significantly decreased recurrent biliary events (p < 0.001), according to research by Reinders et al. [18].

Death Rate Associated With Biliary Sepsis

There was no significant difference in death rates between the early and delayed treatment groups in any of the included studies.

Post-treatment Complications and Adverse Events

While there were no appreciable differences in postoperative complications between early and delayed LC, Riquelme et al. [20] discovered that early LC significantly shortened the length of stay (p = 0.001). Jaundice did not raise the risk of complications in patients undergoing LC, according to Poh et al. [17]. According to El Nakeeb et al. [23], the delayed LC group had noticeably higher morbidity (p = 0.03).

Length of Stay at Hospital

Research has consistently shown that early LC results in shorter hospital stays than delayed LC. For example, stays in early LC groups were found to be significantly shorter in both Akyürek et al. and Riquelme et al. [17,20].

Readmission Rate

Recurrent biliary symptoms and complications were found to be the cause of higher readmission rates in delayed LC groups [20,23].

Quality of Life

The included studies did not directly measure quality of life metrics. On the other hand, shorter hospital stays and fewer recurrent biliary events indicate improved postoperative recovery in early treatment groups.

Comparative review of treatment options

Our systematic review identified several intervention strategies for the management of biliary diseases such as delayed and early LC, CM, and various endoscopic techniques. The following section provides a comprehensive review of each of these approaches in detail.

Delayed Laparoscopic Cholecystectomy (D-LC) Versus Early Laparoscopic Cholecystectomy (E-LC)

Recurrence rates: With a p-value of <0.001, Reinders et al. [18] found that the E-LC group (1 patient, 4.3%) had significantly fewer recurrent biliary events than the D-LC group (17 patients, 36.2%). Comparably, El Nakeeb et al. [23] discovered that, with a p-value of 0.03, there were fewer recurrent biliary symptoms in the early LC group (1 patient) than in the delayed group (7 patients, 12.7%).

Complication rates: E-LC was linked, with a p-value of 0.001, to a significantly shorter length of stay (58 hours) than D-LC (167 hours), with no discernible differences in the need for ERCP. This was reported by Riquelme et al. [20].

Success rates: Intraoperative ERCP and LBDE did not significantly differ in duct clearance rates (87% vs. 69%), according to Poh et al. [27]. However, the ERCP group had lower retained stone rates (15% vs. 42% in LBDE), highlighting its procedural efficacy.

*Conservative Versus ES* *Treatment*

Vracko et al. discovered that ES enhanced 48 patients' clinical outcomes and lessened the need for an emergency cholecystectomy, with three patients (2.8%) experiencing iatrogenic complications [19].

Combining Laparoscopic and Endoscopic Methods

Success rates: In endoscopic stone removal, Iodice et al. reported a 94% success rate without significant ERCP or surgical complications [22].

Complications and length of hospital stay: When comparing laparoscopic rendezvous to preoperative ES, Morino et al. [25] found significantly higher CBD clearance rates (95.6%) and shorter hospital stays (4.3 days vs. 8.0 days, p < 0.0001).

Patient Outcomes and Economic Effectiveness

Hospital stay and costs: Compared to delayed LC, Akyürek et al. showed that early LC after percutaneous cholecystostomy led to shorter hospital stays (mean difference of four days) and lower costs [17].

Operational difficulties: Impacted stones and sphincter of Oddi stenosis were found to have an impact in Liu et al. with primary closure procedures in LC. The only factor that significantly differed between the two treatment groups was the location of the stone [24].

By reducing recurrent biliary events, shortening hospital stays, and lowering healthcare costs, early LC after initial management of biliary sepsis - either through percutaneous cholecystostomy or ES - significantly improves clinical outcomes. Between early and delayed LC, there were no significant variations in death rates or serious postoperative complications. These findings support the early intervention approach to effectively manage biliary sepsis and enhance patient prognosis.

Discussion

The findings cover 14 primary studies, cohorts, and RCTs, which offer important information about the long-term consequences of biliary sepsis in patients treated with cholecystectomy. This is in comparison to CM after sepsis from post-cholecystitis and post-cholangitis. The combined data revealed several major themes that addressed different variations of treating biliary sepsis and its aftereffects. Our systematic review results indicate that when compared to CM and delayed LC, early LC significantly improves long-term outcomes in patients with biliary sepsis. In numerous studies, early intervention consistently decreased the frequency of recurrent biliary events and shortened hospital stays, which reduced healthcare costs and improved patient recovery. The clinical benefits of early LC are highlighted by its lower morbidity and fewer complications, even though there was no significant difference in mortality rates between the two treatments. These findings support earlier research that emphasized the need for timely LC in lowering the risk of sepsis recurrence and enhancing overall prognosis when treating acute cholecystitis and cholangitis [17]. Consistent with findings from multiple studies, early LC demonstrates advantages in reducing recurrent biliary events, decreasing the length of hospital stays, and lowering healthcare costs, particularly in patients managed initially with percutaneous cholecystostomy or ES. Studies like Akyürek et al. [17] and Reinders et al. [18] indicate these benefits in high-risk patients, while Riquelme et al. [20] demonstrate similar outcomes for patients with MGP, suggesting the broad applicability of early LC. Together, these results support early intervention as a beneficial approach for various biliary disease contexts.

Our study also highlights that percutaneous cholecystostomy (PC) has a central role in treating sepsis in patients who are at high surgical risk. In addition, early LC can be safely carried out to lower the risk of recurrent sepsis once acute infection and sepsis have cleared. By removing obstruction at the CBD level, ES also seems to be beneficial in improving clinical outcomes for elderly patients suffering from acute cholecystitis and may reduce the risk of biliary sepsis [17-20]. It also investigated how well conservative oxygen therapy worked for sepsis patients in ICUs. Despite the lack of a statistically significant decrease in mortality when compared to standard oxygen therapy, this finding needs more research and application in clinical settings [21-23]. In terms of intervention timing, papillotomy, and early endoscopic retrograde cholangiopancreatography (ERCP) did not considerably lower the risk of obstructive jaundice in patients suffering from acute biliary pancreatitis. Nevertheless, delaying LC after ES may cause recurrent biliary events in a considerable number of patients, indicating the possible advantages of starting LC as soon as 72 hours after sphincterotomy [24-27]. Additionally, the review examines different surgical techniques, such as laparoscopic procedures and combined endoscopic-laparoscopic strategies, for the management of gallbladder stones and choledocholithiasis. These methods seem safe and efficient for removing stones, with some requiring less time in the hospital and costing less than others. The laparoscopic technique was noted as more effective, successful, and linked to fewer needless procedures. However, more investigation is required to completely comprehend the best management plans for patients suffering from biliary sepsis and related complications [28-31].

Despite undergoing ERCP, many elderly patients with common bile duct (CBD) stone disease continue to have biliary symptoms and complications [32]. For these patients, the study suggests risk-reducing cholecystectomy after ERCP, especially if comorbidities do not prohibit surgery [33]. In clinical practice, surgeons appear to carefully choose patients with shorter life expectancies for CM. Many of these patients continue to have gallstone-related symptoms repeatedly throughout their lives, even though CM may be appropriate for some of them. This emphasizes the difficulties in managing CBD stone disease in elderly patients and how crucial it is to take each patient's preferences and unique circumstances when choosing the best course of action [34]. According to a study by Stefanova et al. [35], advanced age alone should not be a reason for surgery when it comes to treating gallstone pancreatitis in elderly patients. Cholecystectomy is still the recommended course of treatment. Despite worries about surgical risks in the elderly, the study indicates that age should not be the only criterion used to evaluate a patient's suitability for surgery for gallstone pancreatitis. The importance of timely and appropriate management of biliary conditions in elderly patients is highlighted, as it can prevent adverse outcomes and improve overall quality of care. It also emphasizes the significant impact of biliary complications in the elderly population, which can result in high rates of re-admission, mortality, and morbidity [35]. Bergeron et al. highlight the importance of prompt cholecystectomy after ERCP for gallstone disease and CBD clearance [36]. It recommends that cholecystectomy be carried out as soon as the patient is judged suitable for surgery, preferably within the first few days following hospital admission, since postponing it beyond seven days greatly raises the risk of recurrent biliary events. This is further supported by Friis et al. [37], which suggests that LC within 24 hours of ERCP is effective in reducing conversion rates with fewer complications and reduced recurrence rates. Thus, regarding the management of gallbladder and CBD stones, prompt surgical intervention is essential to reduce complications and enhance patient outcomes.

In summary, the present systematic review highlights the significance of timely intervention, suitable surgical methods, and customized patient care in enhancing clinical results and mitigating complications linked to biliary sepsis. The findings highlight that early LC, combined with either PC, ES, or ERCP, not only effectively relieves symptoms but also reduces the risk of recurrent biliary events and complications. Unlike previous studies revolving around specific pathologies leading to acute biliary presentations such as pancreatitis, our systematic review provides comprehensive evidence of surgical interventions available for the management of a range of acute biliary presentations including cholecystitis, cholelithiasis, choledocholithiasis, cholecystocholedocholithiasis, and gall stone pancreatitis. Our study can contribute to the development of evidence-based guidelines for the management of acute biliary diseases. Further research is warranted to refine patient selection criteria and optimize postoperative care.

This systematic review has some strengths and limitations. The strengths of this systematic review consist of using a comprehensive search methodology, including multiple electronic databases such as PubMed, and Cochrane Library, to ensure wide coverage of relevant literature on biliary sepsis management. This was further enhanced by including grey literature sources such as conference proceedings, dissertations, and clinical trial registries, capturing studies not typically indexed in major databases. Furthermore, backward, and forward citation tracking was employed to examine reference lists of included studies and identify newer articles citing them, thus broadening the scope. The review also included a variety of study designs, such as RCTs, cohort studies, case-control studies, and observational studies, allowing for a comprehensive view of biliary sepsis management. This inclusion of diverse study types provided multiple perspectives and methodologies, contributing to a more robust review of outcomes. Moreover, a standardized data extraction protocol was used to ensure consistency and reduce bias in data collection, while quality assessment tools like CASP criteria were employed to ensure the evidence synthesized was of high quality. These strategies ensured thorough coverage of relevant literature and provided a comprehensive review of the long-term effects of cholecystectomy versus CM for biliary sepsis. However, limitations exist, such as heterogeneity among the included studies, individual study analysis, and the possibility of bias.

To address the limitations of individual study analysis, we must consider both the specific populations studied and the available evidence from which conclusions can be drawn. However, given the current scarcity of studies that offer pooled data, our recommendations are based on individual studies, which may limit the generalizability of our findings to broader patient populations. By not combining data across multiple studies, there is a risk of under-representing critical outcomes, such as mortality and long-term morbidity, which might benefit from pooled analysis. Future studies could build on this work by conducting meta-analyses that integrate data across studies, creating a more cohesive perspective on outcomes like LOS, complication rates, and cost-effectiveness. Such an approach would provide a more definitive guide for clinical decision-making in biliary sepsis management. Enhanced study planning and statistical adjustments could further clarify the impact of early versus delayed intervention in reducing biliary complications.

Nevertheless, confounders present in each study were identified after thoroughly assessing included studies and extracted data with their potential implications on the reliability of results. The review’s reliance on English-language publications might have also introduced language bias by excluding relevant non-English studies. Despite these boundaries, the review provides valuable insights into biliary sepsis management, assisting clinicians in decision-making and highlighting areas requiring further research and development.

## Conclusions

This systematic review comprehensively evaluated the long-term consequences of biliary sepsis in patients undergoing cholecystectomy in comparison to CM following post-cholecystitis and post-cholangitis sepsis. Numerous key findings have emerged through the synthesis of evidence from existing literature. Cholecystectomy remains a cornerstone in the control of biliary sepsis with favorable long-term effects, consisting of decreased recurrence rates and advanced overall survival. However, CM may still be suitable for selected patients, particularly those at high surgical risk or with significant comorbidities. Our review underscores the importance of individualized treatment strategies tailored to patient characteristics and preferences. Implications for future practice include the need for clinicians to carefully assess patient profiles when deciding between surgical and CM of biliary sepsis. Additionally, our study highlights the suboptimal long-term outcomes and diminished quality of life among patients managed conservatively. These findings highlight the need for further research to optimize these treatment strategies.
